# Three new species of Entomobryidae (Collembola) from Romania, including a synanthropic *Willowsia* species

**DOI:** 10.3897/zookeys.1282.194867

**Published:** 2026-06-12

**Authors:** Rafael Jordana, Cristina Fiera, Enrique Baquero

**Affiliations:** 1 Universidad de Navarra, Facultad de Ciencias, Departamento de Biología Ambiental, c/ Irunlarrea 1, 31008-Pamplona, Navarra, Spain Universidad de Navarra, Facultad de Ciencias, Departamento de Biología Ambiental Pamplona Spain; 2 Institute of Biology Bucharest, Romanian Academy, Bucharest, Romania Universidad de Navarra, Instituto de Biodiversidad y Medioambiente BIOMA Pamplona Spain; 3 Universidad de Navarra, Instituto de Biodiversidad y Medioambiente BIOMA, c/ Irunlarrea 1, 31008-Pamplona, Spain Institute of Biology Bucharest, Romanian Academy Bucharest Romania

**Keywords:** Chaetotaxy, *
Entomobrya
*, springtails, synanthropic, taxonomy

## Abstract

This study describes three new species of Palaearctic Collembola—*Entomobrya cezarae***sp. nov**., *E. antoniae***sp. nov**., and *Willowsia solanella***sp. nov**.—collected in Romania. The genus *Entomobrya*, one of the most diverse and taxonomically challenging groups of Collembola, has long been affected by incomplete historical descriptions. Recent advances in macrochaetotaxy-based methodologies, particularly those developed by Jordana and collaborators, have significantly improved species diagnoses and clarified the taxonomy of the tribe Entomobryini. In addition, recent global revisions further emphasize the need for standardized morphological terminology. The genus *Willowsia*, comprising 44 species with a worldwide distribution and characterized by distinctive scales, is here supplemented by the description of a new species. These findings contribute to a more robust understanding of the diversity of Palaearctic Entomobryidae.

## Introduction

*Entomobrya* Rondani, 1861 is one of the largest and most taxonomically complex genera within Collembola, currently comprising more than 350 species worldwide. Many taxa remain poorly known due to incomplete old descriptions, often based on colour pattern or fragmentary macrochaetotaxic information. This has complicated species identification and has led to uncertain distributional records. In 2005, Jordana and Baquero (2005) proposed a methodology to identify the minimal set of macrochaetae required for reliable species diagnosis, based on extensive material of *Entomobrya schoetti* Stach, 1922 (Jordana and Baquero 1999). Subsequently, with the collaboration of numerous museums and researchers, the Palaearctic members of the tribe Entomobryini were comprehensively revised (Jordana 2012). This work complemented earlier studies from the United States by Peter Bellinger and Kenneth Christiansen (Christiansen and Bellinger 1980), which relied primarily on general morphology and colouration for species identification. More recent revisions, including those by Jordana and Greenslade (Jordana and Greenslade 2020) in Australia, and by Viana et al. (2024) on the Amazonian fauna of Brazil, have further clarified the taxonomy of this globally distributed genus, highlighting the need for modern diagnoses that integrate detailed dorsal macrochaetotaxy in species diagnoses and standardized morphological terminology.

The genus *Willowsia* Shoebotham, 1917 currently includes 44 species (Bellinger et al. 1996–2024) and has a worldwide distribution. Some species are frequently reported from indoor environments (Cipola and Katz 2021). Although morphologically similar to *Entomobrya*, *Willowsia* species are distinguished by the presence of characteristic body scales.

This study reports three new Palaearctic Collembola species—*Entomobrya cezarae* sp. nov., *Entomobrya antoniae* sp. nov., and *Willowsia solanella* sp. nov.—based on material collected in Romania by Cristina Fiera between 2008 and 2012. The aim of this study is to describe these three new species, providing detailed morphological and chaetotaxic analyses to support their diagnosis and differentiation from related taxa. The present paper provides a full description of the new species, covering morphology, chaetotaxy, diagnostic features, and comparison with related species. Notes on habitat, synanthropic behaviour, and ecological significance of these three species are also included.

## Material and methods

During routine examination of microarthropods associated with household environments, a distinct species of *Willowsia* was discovered in a cardboard box containing potatoes in an apartment in Bucharest, Romania. A detailed morphological analysis, including the chaetotaxy of the head, thorax, and abdomen, as well as the structure of the distal antenna, furca, claw complex, and body scales, revealed a unique combination of characters not corresponding to any previously described species.

The condition of the specimens—very soft and with most lacking complete legs or antennae—made their description difficult, and some characters could not be observed. After preliminary sorting, some specimens of each species were mounted in Hoyer’s medium for observation under a microscope (some specimens were cleared in Nesbitt’s fluid). The remaining samples were preserved in 70% ethyl alcohol. The slides were examined using two microscopes: an Olympus BX51 TF (Olympus Group, Tokyo, Japan), equipped with phase contrast, and a Zeiss Axio Imager A1 with differential interference contrast (DIC). Measurements were taken using a U-DA drawing attachment UIS (Universal Infinity System) and a scale calibrated with a slide from Graticules Ltd, Cambridge, UK (1 mm divided in 100 parts).

The nomenclature follows Fjellberg (1999) for the labial palp, Gisin (1967) for labial chaetae, and Jordana and Baquero (2005) for the dorsal macrochaetotaxy of the head and tergites, including the formula proposed for dorsocentral macrochaetotaxy of Th II, and Abd II–IV, and Soto-Adames (2008), both derived from Szeptycki (1979). Types of chaetae and sensilla follow Deharveng (1983) and Jantarit et al. (2020). The terminology of scales in *Willowsia* follows Zhang et al. (2011).

The type material has been deposited in the Zoology Museum of the University of Navarra (**MZNA**).

### Abbreviations

**Mc** macrochaeta-ae;

**ms** microsensillum-a;

**as** anterior sensilla;

**Abd** Abdominal tergite;

**mes** mesochaeta-ae;

**mic** microchaeta-ae;

**Ant** Antenna;

**Th** Thoracic tergite;

**MZNA** Museo de Zoología, Universidad de Navarra.

## Results

### Class Collembola Lubbock, 1873

**Order Entomobryomorpha Börner, 1913**

**Family Entomobryidae Tömösvary, 1882**

#### Genus Entomobrya

Taxon classificationAnimaliaDiplostracaEntomobryidae

Rondani, 1861

13413F3F-A958-5A73-B8AC-89D555D1BCEC

##### Type species.

*Degeeria muscorum* Nicolet, 1842: 75.

#### Entomobrya cezarae sp. nov.

Taxon classificationAnimaliaDiplostracaEntomobryidae

9E2CBC19-BC96-5399-BB15-2919EDF2E764

https://zoobank.org/4E4287CC-860F-4DE1-A07F-F96A084317EA

Figs 1, 4–12, Table 1

##### Type material.

***Holotype*** • ♀ on slide, **Romania**, Singureni locality, Giurgiu County, rape crop, Barber traps, 18-V-2011, 44°13'48.2"N, 25°56'53.7"E, 65 m a.s.l., sample code IBB-ROSIN-05.11 (MZNA 808373), C. Fiera leg.

**Figures 1–3.**
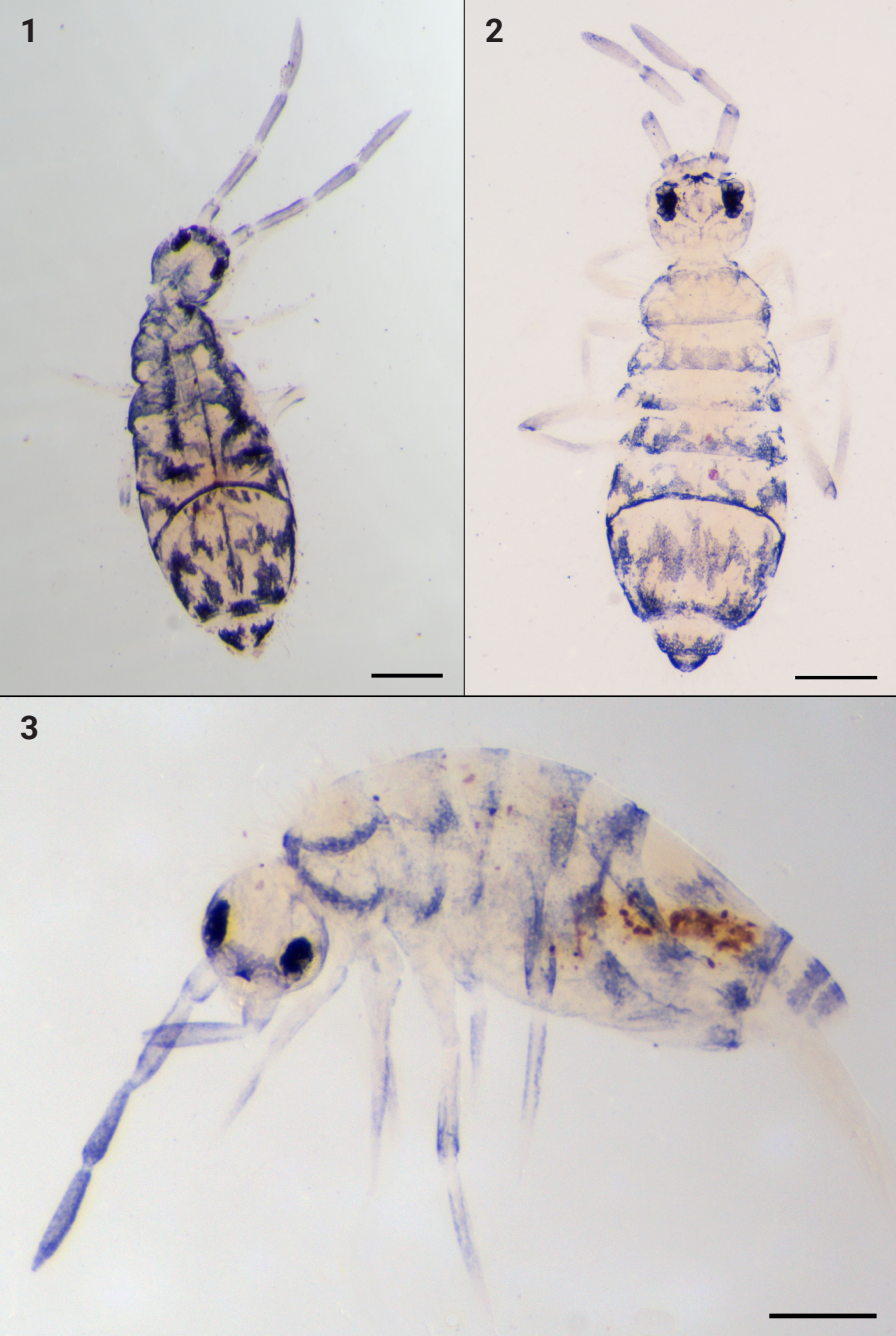
Habitus of the new *Entomobrya* and *Willowsia* species. **1**. *E. cezarae* sp. nov. (holotype); **2**. *E. antoniae* sp. nov. (holotype); **3**. *W. solanella* sp. nov. Scale bars: 0.25 mm (**1, 2**); 0.1 mm (**3**).

**Table 1.** Comparison of species sharing the same macrochaetotaxy on Abd II–III based on 26 morphological and chaetotaxy characters used for species differentiation.

Characters	Species							
	cez	che	han	hir	kuz	paz	str	tai
H1	4	3*	3*	3–4	3*	2*	4	3*
H2	1	1	1	1	1	2*	2*	3*
H3	0	0	0	0	0	0	0	0
H4	2	3*	3*	3*	3*	3*	3*	3*
H5	2	2	2	1b*	2	3*	2(3)	2
L1	4	3*	3*	1*	3*	3*	2*	U
AV	1	U	3*	2-3*	1	1	2*	1
AL	0.77	U	1.20	1.45	0.90	1.93	1.18	1.30
T1	3	4*	4*	4*	6*	8*	3	6*
T2	3	6*	6*	5*	6*	5*	5*	6*
E1	1	0*	0*	1	0*	0*	0*	0*
TH	1	1	1	1	1	1	2*	1
A1	2	2	2	2	2	2	2	2
A2	5	6(5–7)	5	4–5	5	5	5(7)	5
A3	0	0	0	0	0	0	0	0
A4	2	2	2	2	2	2	2	2
A5	2	2	2	2	2	2	2	2
A6	0	0	0	0	3*	9*	0	0
A7’	0	0	0	0	0	1*	0	0
A7	4	7(11)*	3*	5*	4	6*	3*	6*
A8’	0	0	0	1*	0	1*	0	0
A8	5	7(10)*	4*	4*	3*	4*	3*	1*
A9	2	3(5)*	3*	2(3)	4*	5*	3*	5*
A10	2	8(10)*	2	3*	2	3*	2	3*
MC	5	5	4*	13–15*	7*	11*	4*	5
MP	2	2	2	2	2	2	2	2
D	—	10	11	11	10	16	11	10
%	—	42	42	42	38	62	42	40

Legend for columns headers: *cez*, *E. cezarae* sp. nov.; *che*, *E. cheni*; *han*, *E. handschini*; *hir*, *E. hirsutothorax*; *kuz*, *E. kuznetsovae*; *paz*, *E. pazaristei*; *str*, *E. strigata*; *tai*, *E. taigicola*. Legend for the rows: Head: H1, number of Mc in series sd’4–sd’4' (An2-3); H2, number of Mc in series sd4–sd’3a (A5, A7); H3, number of Mc in series d’0 (S’0); H4, number of Mc in series d1, sd1, sd’1 (S1, S3, S4); H5, number of Mc in series v1, v3, v4 (Ps2, Ps3, Ps5); L1, labral papilla presence and shape: absent (1), simple-smooth (2), multispinose (3), chaeta like projection (4); AV, antennal vesicle shape: absent (0), lobule simple (1), bilobed (2), trilobed (3); AL, antennal length, mm. Th II: T1, number of Mc in series m1, m2i2 (coded as 5 if > 4); T2, number of Mc in series a5, m5 (coded as 9 if > 8). Leg: E1, empodium, shape of external lamella (pe) of leg III: smooth (0), serrate (1), with tooth (2); TH, tenent hair shape: clavate (1), acuminate (2). Abd II: A1, number of Mc in series a2, a3; A2, number of Mc in series m3 series; A3, number of Mc in series a1; A4, number of Mc in series above m2; A5, number of Mc in series m3, m4 series. Abd IV: A6, number of Mc in series a1, a5 (A1 E1a); (coded as 9 if > 8); A7’, number of unpaired Mc in series ma0 (A03); A7, number of Mc in series ma1 ma4 (A2, E1) (coded as 10 if > 9); A8’, number of unpaired Mc in series m0 (A04); A8, number of Mc in series m1, m3 (A4p, C4) (coded as 6 if > 5); A9, number of Mc in series mp1, mp3 (A5, B5) (coded as 6 if > 5); A10, number of Mc in series p1–p3 (A6i, B6) (coded as 6 if > 5). Furca: MC, number of manubrial plate chaetae; MP, number of manubrial plate pseudopores. *Additional notation*. *, difference from the new species; D, total number of differences between species and the new species; **%**, percentage of differences relative to the new species (based on characters); U, Unknown. Occasional values are given in parentheses.

***Paratypes*** • ♀ on slide, with one additional specimen on the same slide, same data as the holotype (MZNA 808374) • 6 specimens in ethyl alcohol, same data as holotype, sample code IBB-ROSIN-05.11 (MZNA 808383 to 808388) • 2 ♀♀ on one slide, **Romania**, Voineasa locality, Vâlcea County, beech forest, litter, 01-XI-2012, 45°24'32.38"N, 23°58'4.90"E, 630 m a.s.l., sample code IBB-ROVOI-11.12 (MZNA 808375 to 808376). C. Fiera leg.

##### Description.

***Size***: body length (excluding antennae) up to 1.95 mm (holotype), 1.60 mm (*n* = 4).

***Colour pattern***: ground colour white or very pale yellow, with pigment forming five discontinuous longitudinal lines, similar to *E. handschini* Stach, 1922 (Fig. 1).

**Figures 4–9.**
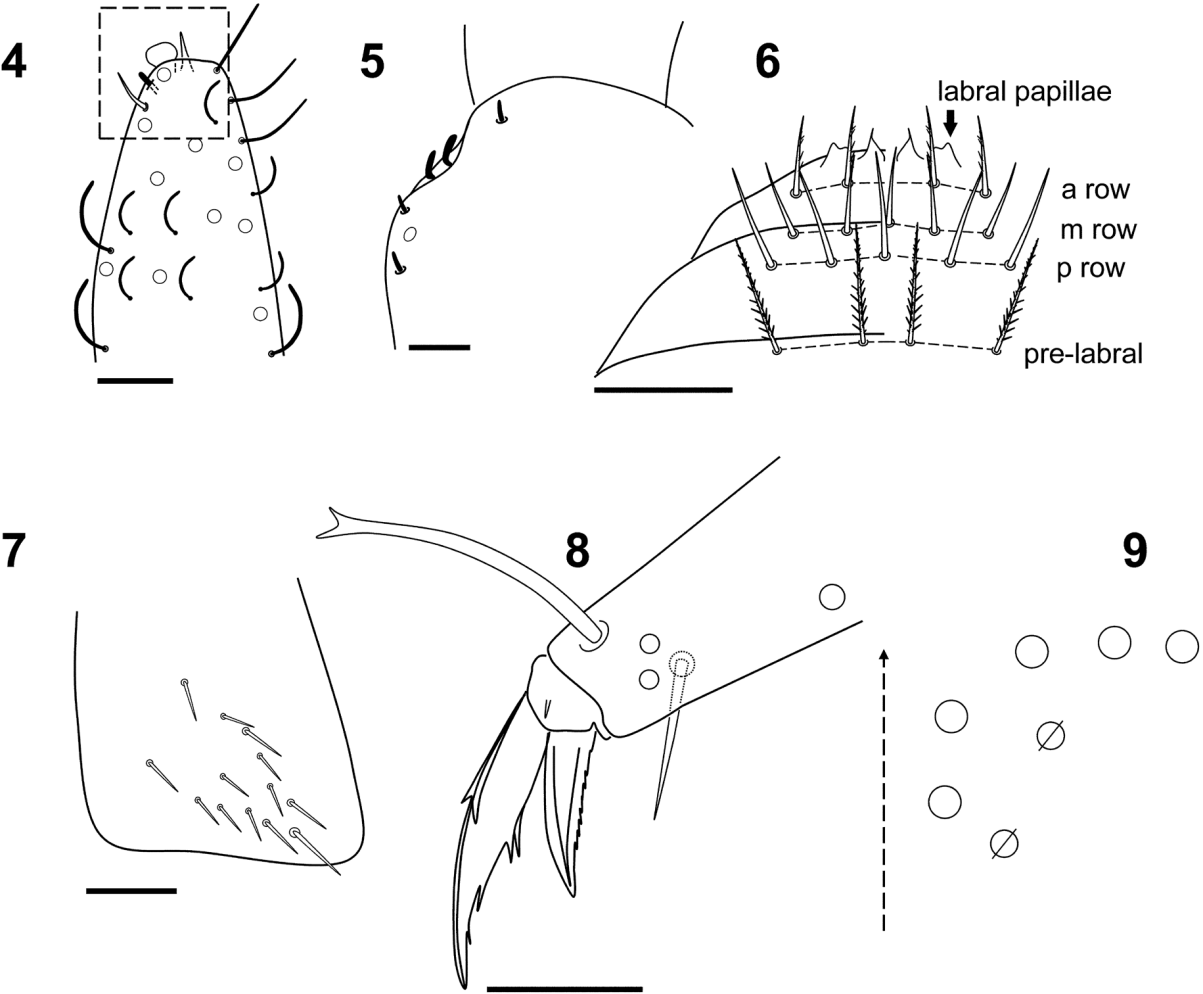
*Entomobrya cezarae* sp. nov. **4**. Tip of the Ant IV; **5**. Ant III sensory organ, with the typical rod sensilla and three guard chaetae; **6**. Labral chaetae and papillae; **7**. Trochanteral organ; **8**. Tip of the leg III; **9**. Manubrial plate. Scale bars: 0.04 mm (**4**); 0.01 mm (**5**); 0.02 mm (**6–8**).

***Head***: eight eyes; GH smaller than EF. Antenna 0.80 mm long, 2.3–3.0× head length (*n* = 4). Ant IV with simple apical vesicle, pin chaeta present (not bifid), and rod-like subapical organite (Fig. 4). Sensory organ of Ant III with the special bent, rod-like sensilla and three additional guard sensilla (Fig. 5). Relative length of Ant I/II/III/IV = 113/210/194/256 (µm) = 1/1.7/0.9/2.3 (*n* = 3). Prelabral chaetae ciliated, labral rows ‘a’, ‘m’, and ‘p’ smooth. Labral papillae unispinose on the two middle papillae (Fig. 6). Lateral process of labial papilla E reaching the tip. Maxillary palp bifurcated, with three sublobal chaetae.

***Body and legs***: length ratio of Abd IV/III = 5 (range 3.22–6.00; *n* = 4). Trochanteral organ with approximately 11–15 chaetae (Fig. 7). Tibiotarsus not sub-segmented, lacking smooth chaetae except for one smooth terminal chaeta on legs III.

Claw with four inner teeth: paired teeth at 50% and first unpaired tooth at 75% from base; dorsal teeth not basal. Empodium lanceolate, with serrated external lamella (pe) on leg III. Tenent hair clavate (Fig. 8). Manubrium and dens lengths 0.26 and 0.37 mm, respectively (mean, *n* = 2). Manubrial plate with five chaetae and two pseudopores (Fig. 9). Mucro with teeth of similar size, mucronal spine reaching tip of subapical tooth; non-crenulated area of dens twice the length of the mucro.

***Macrochaetotaxy***: simplified Mc formula: 4-1-0-2-2/3-3/2-5/0-2-2/0-4-5-2-2.

**Figures 10–12.**
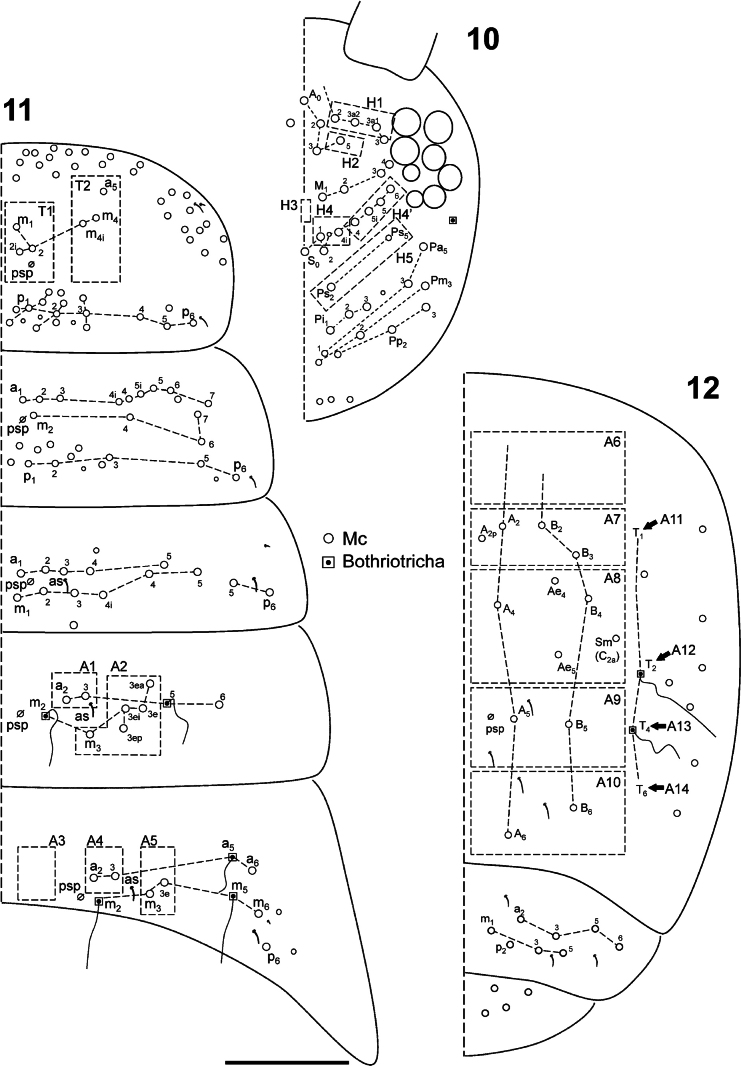
*Entomobrya cezarae* sp. nov. dorsal macrochaetotaxy (see Jordana and Baquero 2005 for the nomenclature of the areas H1–H5, T1–T2, and A1–A14). **10**. Head; **11**. Thorax II–III and Abd I–III; **12**. Abdominal IV and partial Abd V. Scale bar: 0.1 mm.

Head (Fig. 10): H1 area with An_2_, An_3a2_, An_3a1_ and An_3_ as Mc; H2 area with one Mc (A_5_); H4 area with two Mc (S_1_ and S_4i_); H4’ area with four Mc (S_6_, S_5_, S_5i_, and S_4_); H5 area with Ps_2_ and Ps_5_ as Mc. Mesothorax (Fig. 11): T1 area with three Mc (m_1_, m_2_ and m_2i_); T2 area with three Mc (m_4_, m_4i_, and a_5_, without m_5_); metathorax with a_1_-a_7_, m_2_, m_4_ and m_6_–m_7_, p_1_–p_3_, p_5_–p_6_ as Mc. Abdomen (Fig. 12): Abd II A1 area with two Mc (a_2_ and a_3_); area A2 with five Mc, (m_3_, m_3ep_, m_3e_, m_3ei_, and m_3ea_); Abd III without Mc on area A3, and two Mc on A4 (a_2_ and a_3_) and A5 (m_3_ and m_3a_ always present); Abd IV with A_2_, A_2p_, A_4-6_, Ae_4–5_, B_2–6_, and Sm (C_2a_) as Mc, and at least three sensilla present on posterior area. Abd V with three sensilla. Sensillar chaetotaxy typical for the genus.

##### Etymology.

The species epithet *cezarae* is a feminine genitive noun, dedicated to Cezara, one of the twin daughters of the second author.

##### Ecology.

The species occurs in both lowland and montane habitats. At the type locality (Singureni, Giurgiu County), specimens were collected in an agricultural landscape (rape crop), using Barber traps. Additional material was recorded in Voineasa (Vâlcea County), in leaf litter of a beech forest (*Fagus sylvatica*). These records indicate that the species inhabit both disturbed agroecosystems and natural forest-litter environments.

##### Remarks.

Based on dorsal macrochaetotaxy of Abd II–III (simplified formula 2-5/0-2-2), the new species differs from all described *Entomobrya* species except *E. cheni* Baquero, Arbea & Jordana, 2010, *E. handschini* Stach, 1922, *E. hirsutothorax* Jordana & Baquero, 2021 in Baquero et al. (2021), *E. kuznetsovae* Jordana, Potapov & Baquero, 2011, *E. pazaristei* Denis, 1933, *E. strigata* Stach, 1963, and *E. taigicola* Jordana, Potapov & Baquero, 2011 (all sharing four teeth on internal claw). *Entomobrya strigata* differs in having the sub-apical tooth smaller than the apical one. Among the species of this group, the new species is unique in lacking m_5_ chaeta on Th II. Differences between these species and the new species are summarized in Table 1, with divergence ranging from 38% to 67% across 24–26 diagnostic selected characters.

#### Entomobrya antoniae sp. nov.

Taxon classificationAnimaliaDiplostracaEntomobryidae

71183D2E-7685-5FEF-957A-71AC64DE610F

https://zoobank.org/935A7F05-6BE9-4FF0-A1D0-4B5629A1D806

Figs 2, 13–19, Table 2

##### Type material.

***Holotype*** • ♀ on slide, **Romania**, Odorheiu Secuiesc, Harghita County, 02-X-2008, 46°17'53.8"N, 25°17'43.3"E, 581 m a.s.l., orchard, mosses on *Prunus* tree, sample code IBB-ROODH-10.18 (MZNA 808377), C. Fiera leg.

**Table 2.** Comparison among species sharing the same macrochaetotaxy on Abd II–III, based on 26 morphological and chaetotaxy characters commonly used for comparison.

Characters	Species						
	*bar*	*chu*	*cit*	*kaj*	*lad*	*ant*	ant**
H1	3	4*	4(5)*	3	3(4)	3	3
H2	1*	2*	1*	1*	1*	1*	0
H3	0	0	0	0	0	0	0
H4	2*	2*	2*	1*	2*	3*	0
H5	1-3*	2*	3*	2*	1b*	2*	1a
L1	2*	2*	3	2*	2*	3	3
AV	1-2	1	1	2*	2*	1	1
AL	400*	610*	U	740*	U	200	200
T1	0	0	0	0	0	0	0
T2	2(1)	1	1	1	1(3)	1	1
E1	1*	1*	1*	1*	1*	0	0
TH	1	1	1	1	1	1	1
A1	0	1	1	1	1	1	0
A2	1	2*	2*	2*	2*	2*	1
A3	0	0	0	0	0	0	0
A4	0	1	1	1	1	1	0
A5	1	1	1	1	1	1	1
A6	0	0	0	0	0	0	0
A7’	0	0	0	0	0	0	0
A7	0	0	0	0	0	0	0
A8’	0	0	0	0	0	0	0
A8	0	3*	1	3(2)*	3*	1	1
A9	1	2	1	1	2	2	1
A10	2	2	2	2	2	2	0
MC	3-4	4*	U	5*	4*	3	3
MP	2	2	U	2	2	2	2
D	6	10	6	10	9	-	-
%	23	38	26	38	36	-	-

Legend for columns headers: *bar*, *E. barbata*; *chu*, *E. chungseensis*; *cit*, *E. citrensis*; *kai*, *E. kajjairensis*; *lad*, *E. ladakhi*; *ant*, *E. antoniae* sp. nov.; ant**, E. antoniae sp. nov. but considering only the true Mc. Legend for rows: the same as in Table 1.

***Paratypes*** • ♀ on slide (MZNA 808378) • 9 specimens in ethyl alcohol, same locality as holotype (MZNA 808389 to 808397), C. Fiera leg.

##### Description.

***Size***: body length up to 1.53 mm (slide-mounted specimens: 1.12–1.53 mm).

***Colour pattern***: ground colour pale. Eyepatch dark blue. Ant I–IV and tibiotarsi with blue pigment. Body dark blue with variable pigment distribution (Fig. 2).

**Figures 13–16.**
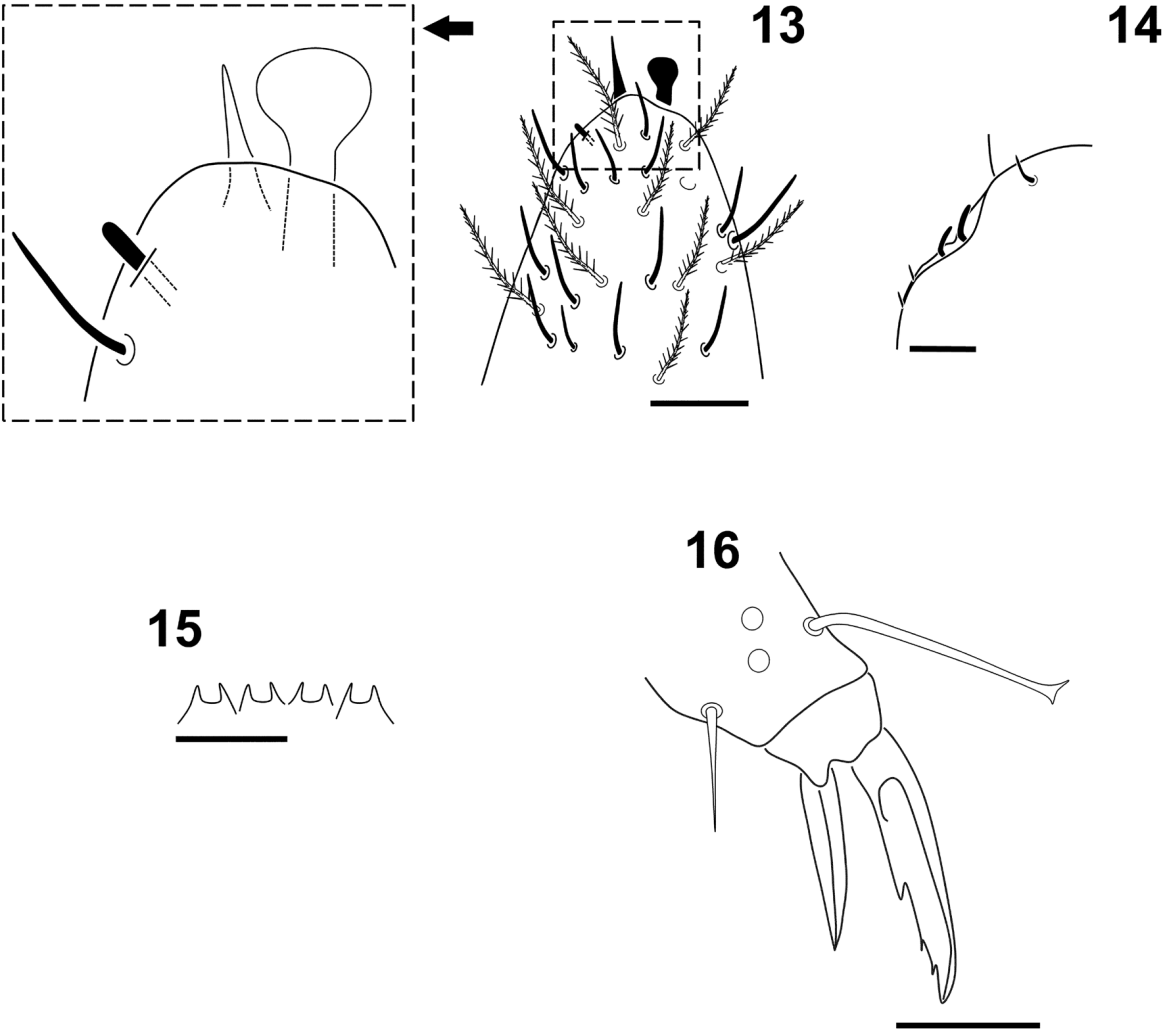
*Entomobrya antoniae* sp. nov. **13**. Tip of the Ant IV, with a magnified view to the left of the antennal tip, showing the terminal vesicle, the pin chaeta, the organite, and its accessory seta; **14**. Ant III sensory organ; **15**. Labral papillae; **16**. Tip of the leg III. Scale bars: 0.01 mm (**13, 14**); 0.02 mm (**15, 16**).

***Head***: antenna/head ratio = 2.07. Relative length of antennal segments I:II:III:IV = 1:3:3.5:3.5 (*n* = 2). Distal part of Ant IV with pointed pin chaetae, and a simple apical vesicle (Fig. 13), subapical organite peg like. Sensory organ of Ant III with two bent, rod-like sensilla, and three guard sensilla (Fig. 14). Eyes 8 + 8, E, F, G and H smaller than anterior ones. Labral chaetae formula 4/5, 5, 4; prelabral and row ‘a’ chaetae ciliated; ‘m’ and ‘p’ rows smooth. Distal margin of labrum with four papillae, each bearing two denticles (Fig. 15). Labial posterior row chaetae finely ciliate (M_2_REL_1_L_2_).

***Body and legs***: length ratio Abd IV/III 3.27–3.45 (*n* = 2). Trochanteral organ with approximately 8–10 spiny chaetae. Tibiotarsus not subdivided, without smooth chaetae except for one smooth terminal chaeta on legs III. Claw with four inner teeth: one paired at 50% of inner edge and two unpaired distal teeth at 70% and 80% from the base. Empodium acuminate, with smooth outer edge. Tenent hair thick, clavate, nearly as long as inner side of claw (Fig. 16). Abdomen. Abd IV 2.6–2.7× as long as Abd III (dorsal midline). Manubrium and dens lengths 0.22 mm and 0.27 mm, respectively (*n* = 2). Manubrial plate with three chaetae and two pseudopores (only one pseudopore observed in one specimen).

Mucro with teeth of similar size, mucronal spine reaching tip of subapical tooth. Non-crenulated portion of dens twice the length of mucro. Sensilla not clearly observed; one microsensillum (ms) present on Abd III.

**Figure 17.**
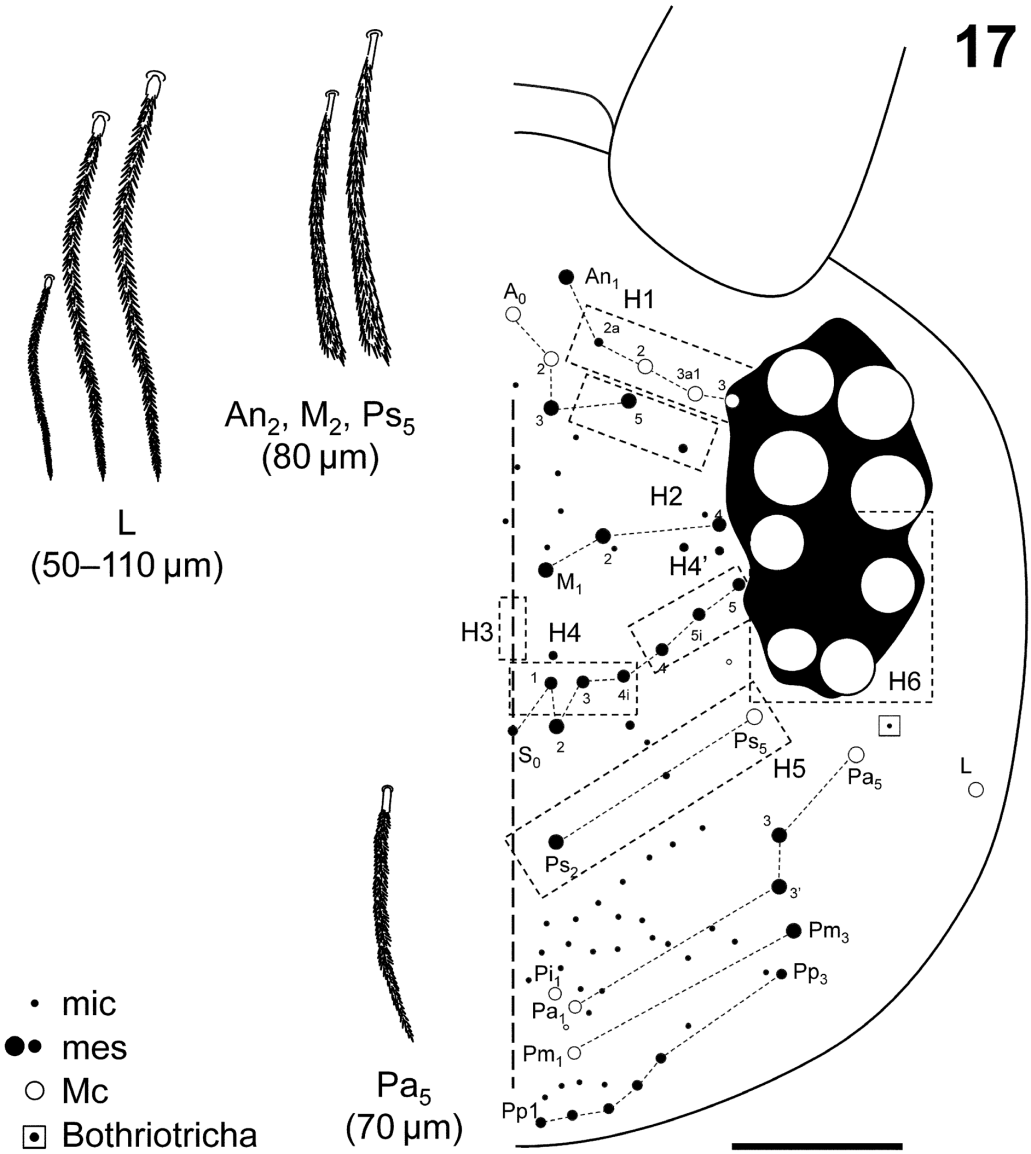
*Entomobrya antoniae* sp. nov. dorsal head macrochaetotaxy. Scale bar: 0.05 mm.

**Figure 18.**
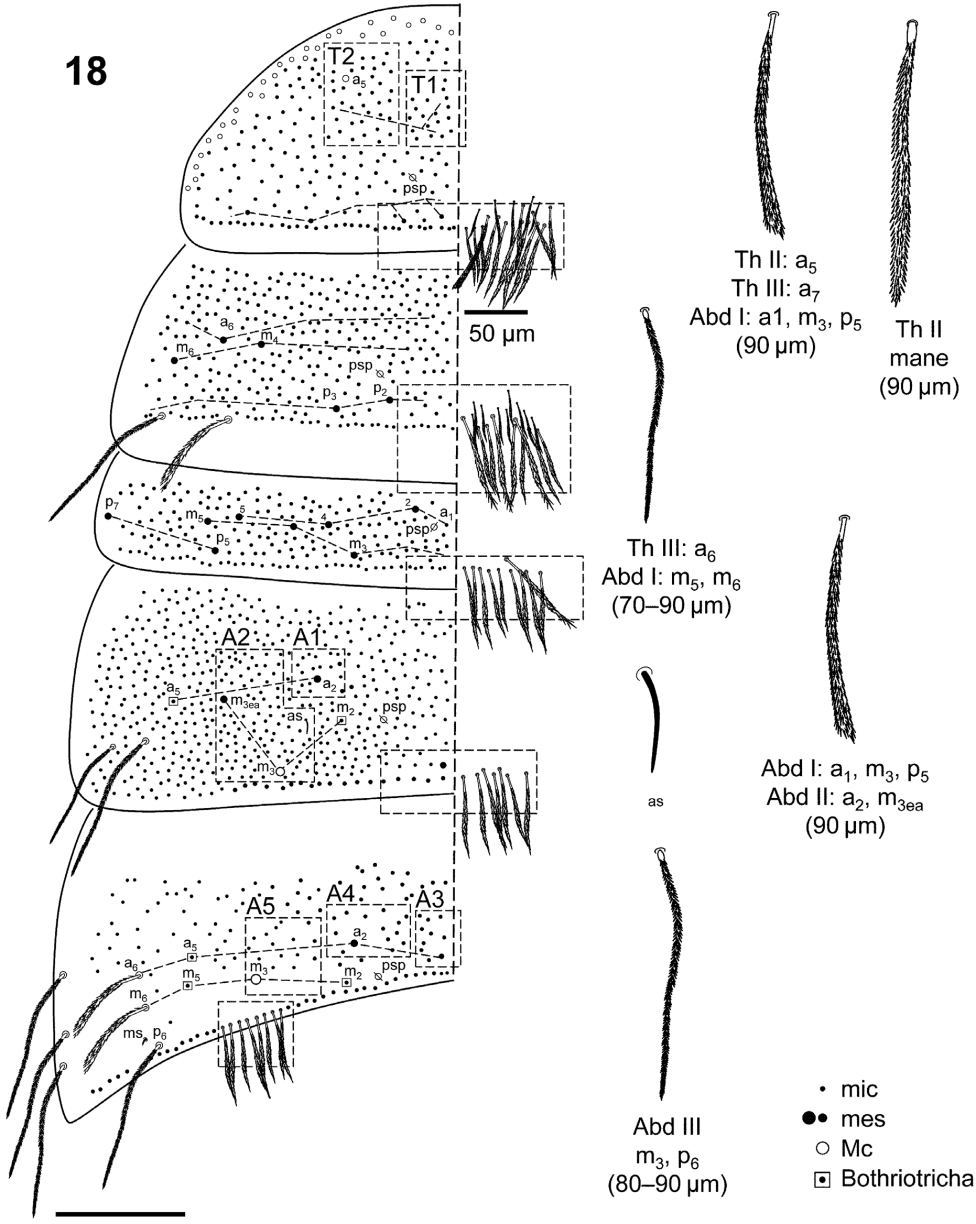
*Entomobrya antoniae* sp. nov. dorsal Th II to Abd III macrochaetotaxy. Scale bar: 0.1 mm.

**Figure 19.**
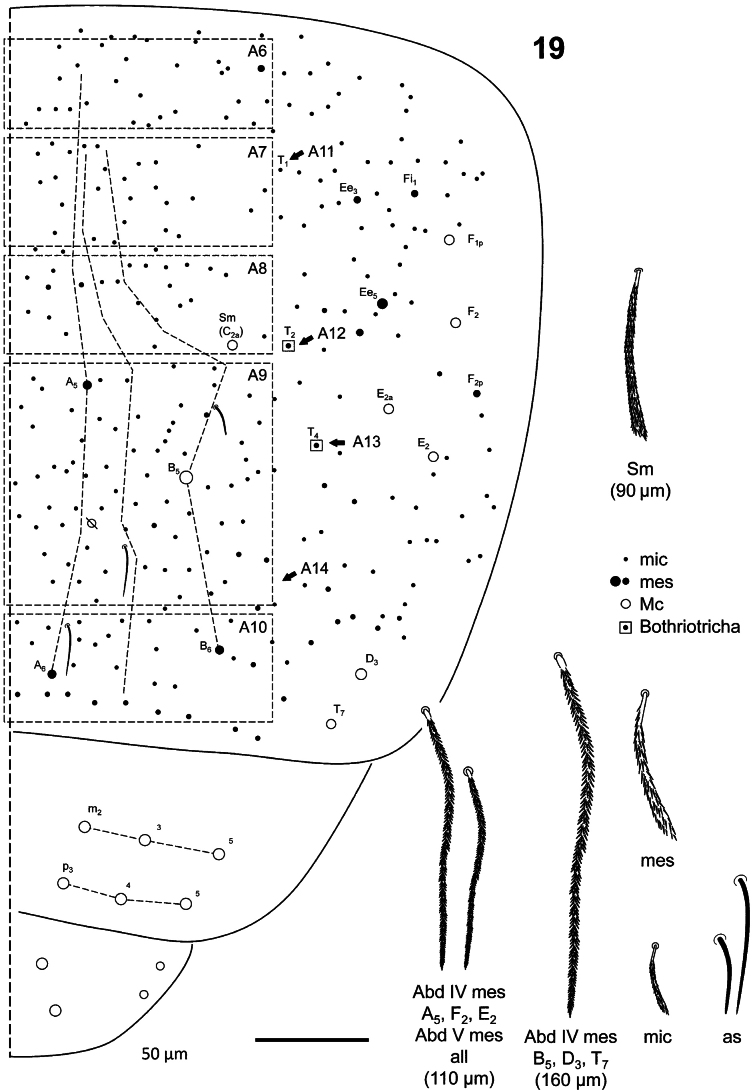
*Entomobrya antoniae* sp. nov. dorsal Abd IV and partial Abd V macrochaetotaxy. The insertion of the macrochaetae is represented in proportion to their size. Scale bar: 0.05 mm.

***Macrochaetotaxy***: limits between meso- and macrochaetae unclear in some cases due to size of specimens. Presence of Mc is indicated, although some may correspond to mesochaetae (mes). Head (Fig. 17): dorsal chaetotaxy: An_1_, An_2a_, An_2_, An_3a1_, An_3_, A_0_, A_2_, A_3_, A_5_, M_1_−M_4_ (M_4_ as mes), S_0_, S_1_, S_2_, S_3_, S_4i_, S_4_, S_5i_, S_5_, Ps_2_, Ps_5_, Pa_3’_, Pa_3_, Pa_5_ and Pm_3_ as Mc; S_0’_ absent. Thorax: Th II with one medio-lateral Mc (a_5_) and main chaetae as Mc; four posterior mes on each side and m_4_ as mes. Specialized S-chaetae and microsensillum (ms) on antero-lateral margin not observed. Th III without central Mc or mes; only three latero-anterior and two centro-posterior small chaetae Mc or mes (a_6_, m_4_, m_6_, p_2–3_). Abdomen: Abd I with a_1–2_, a_4–5_, m_3–5_ and p_5–7_ as small Mc or mes. Abd II with two mes between bothriotricha m_2_ and a_5_ (a_2_ and m_3ea_), m_3_ as Mc, two postero-lateral mes (possibly m_5_ and m_6_) and typical sensilla (as). Abd III with one dorso-central Mc (m_3_), four mes (a_2_, a_6_, m_6_, p_6_), and some lateral large mes or Mc (pointed) (Fig. 18). Abd IV with five large mes or Mc: Sm (C_2a_), A_5–6_, B_5–6_ on central area (A_5-6_ and B_6_ smaller than the other); laterally with long-pointed Mc (Fig. 19).

General chaetotaxy formula: 3-1-0-3-2/0-1/1-2/1-1-1/0-0-1-2-2 (after Jordana and Baquero 2005). If small Mc are considered mes: 3-0-0-0-1a/0-1/0-1/0-0-1/0-0-1-1-0.

##### Etymology.

The species epithet *antoniae* is a feminine genitive noun, dedicated to Antonia, one of the twin daughters of the second author.

##### Ecology.

The species was collected from humid microhabitats consisting of moss cushions growing on the bark of a *Prunus* tree in an orchard. These shaded bryophyte patches retain moisture and represent typical microhabitat for Entomobryinae. The species is currently known only from the type locality in Odorheiu Secuiesc, Harghita County, Romania.

##### Remarks.

Among *Entomobrya* species with known macrochaetotaxy, none exhibit the condition 0-1 (only a_5_) macrochaetae on Th II. If mesochaetae are considered as macrochaetae, the new species shows the formula 0-1/1-2/0-1-1 for Th II and Abd II–III. This combination is shared by only four species: *E. chungseensis* Baquero & Jordana, 2008; *E. citrensis* Katz & Soto-Adames, 2015 (in Katz et al. 2015); *E. kajjairensis* Baquero & Jordana, 2015 (in Baquero et al. 2015); and *E. ladakhi* Baquero & Jordana, 2014 (in Baquero et al. 2014). However, *E. antoniae* sp. nov. differs from these in the chaetotaxy of Abd IV and head, as well as in legs and antennal characters.

Considering only the “true” Mc, a single species, *E. barbata* Siqueira & Bellini, 2020 (in Santos et al. 2020), shares the same Th II and Abd II–III chaetotaxy (0-1/0-1/0-0-1) but differs in head and Abd IV chaetotaxy and in empodium shape. Differences between these species and the new taxon are summarized in Table 2.

#### Genus Willowsia

Taxon classificationAnimaliaDiplostracaEntomobryidae

Shoebotham, 1917

C74A8713-BC6D-52EF-AD0F-F2BDB130BA45

##### Type species.

*Seira nigromaculata* Lubbock, 1873: 146.

#### Willowsia solanella sp. nov.

Taxon classificationAnimaliaDiplostracaEntomobryidae

8514C9F3-7065-53B4-AB85-FC8527576B70

https://zoobank.org/A4CE71EA-D23B-4F52-A6D0-FAA6F38C7DEE

Figs 3, 20− 27, Table 3

##### Type material.

***Holotype*** • ♀ on slide, **Romania**, Bucharest, indoor domestic habitat (collected from a cardboard box containing potatoes), 7-VII-2010, 44°25'48.4"N, 26°09'00.4"E, 74 m a.s.l., sample code IBB-ROBUC-07.10 (MZNA 808379), C. Fiera leg.

**Figures 20–24.**
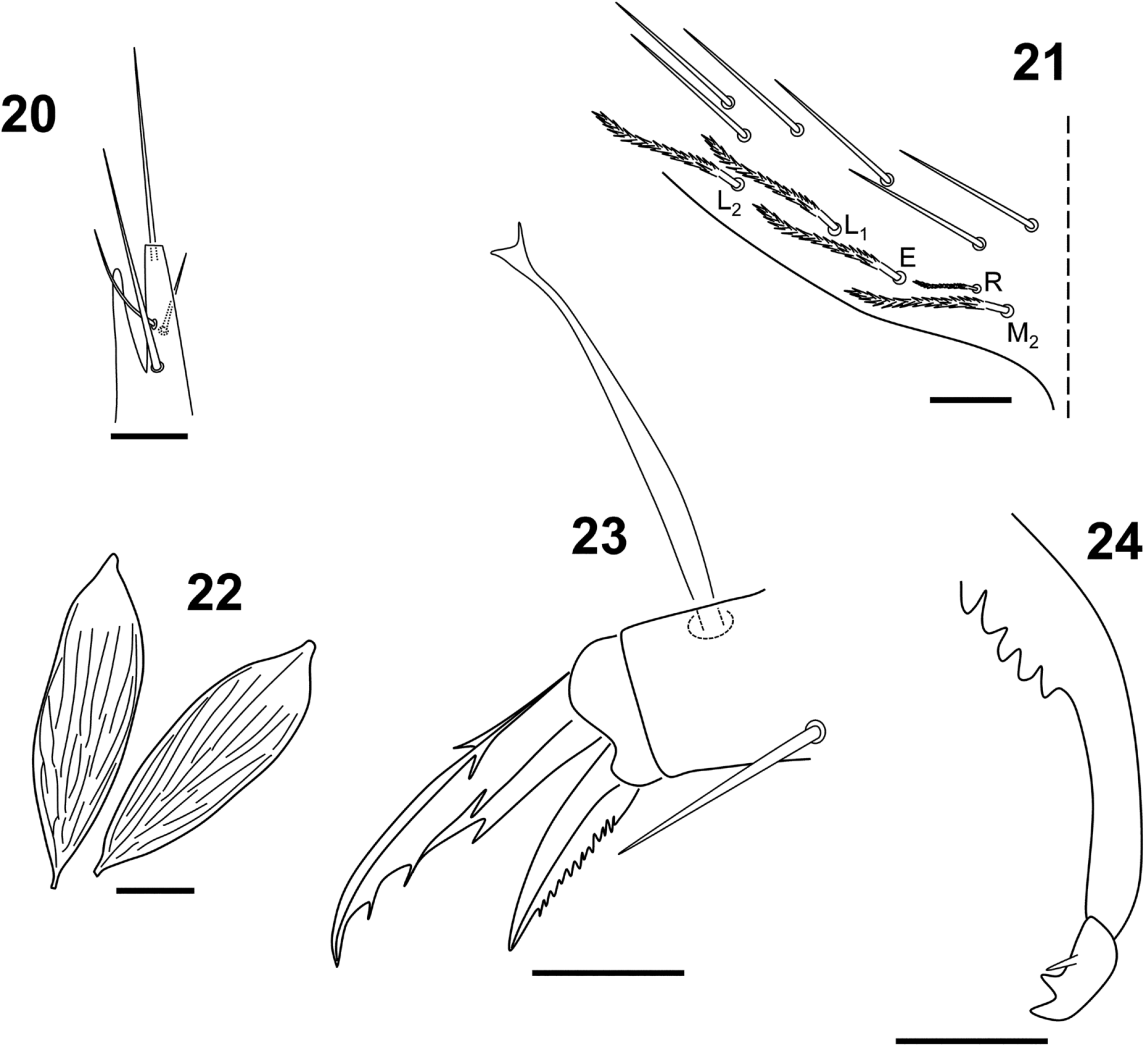
*Willowsia solanella* sp. nov. **20**. Labial papilla E; **21**. Labial chaetae; **22**. Scales, showing ornamentation; **23**. Tip of the leg III; **24**. Tip of dens and mucro. Scale bars: 0.01 mm (**20, 22**); 0.02 mm (**21, 23, 24**).

**Table 3.** Comparison of dorso-central macrochaetotaxy among *Willowsia* species with a similar number of Mc on Abd II–III.

Characters	*W. solanella* sp. nov.	*W. samarkandica*	*W. yiningensis*	*W. zhaotongensis*
		(sensu Zhang et al. 2011)		
Head An Mc (H1)	2	4*	3*	5(6)*
Head A Mc (H2)	1	1	0?	1
Head S_0_	absent	present*	present*	present*
Head S Mc (sutural) (H4)	S_2_–S_5_	S_2_–S_5_	S_1_–S_5_*	S_1_–S_6_*
Head Ps Mc (H5)	Ps_5_	Ps_5_	Ps_1_, Ps_5_*	Ps_1_, Ps_5_*
Th II central Mc (T1)	3	4*	2*	3
Th II lateral Mc (T2)	4 (with m_5_)	3 (no m_5_)*	3 (no m_5_)*	5 (with m_5_)*
Abd I Mc number	3	7*	4*	4*
Abd II (A1)	a_2_–a_3_	a_2_–a_3_	a_2_–a_3_	a_2_–a_3_
Abd II (A2)	m_3_, m_3ep_, m_3ea_	m_3e_, m_3ep_, m_3e_*	m_3_, m_3ep_, m_3e_*	m_3_, m_3ep_, m_3e_*
Abd III	a_2_, a_2a_, m_3_	a_2_, a_2a_, m_3_	a_2_, a_2a_, m_3_	a_2_, a_2a_, m_3_
Abd IV Mc number	16	32*	14*	22*
D	—	6/12	9/12	8/12
%	—	50	75	67

Legend: *, difference from the new species; D, number of differences between each species and the newly described species; %, percentage of differences for each species relative to the new species, based on the total number of comparable characters; U, unknown.

***Paratypes*** • 3 ♀ on slide (MZNA 808380 to 808382) • 9 specimens in ethyl alcohol, all from the same locality as the holotype (MZNA 808399 to 808407), C. Fiera leg.

##### Description.

***Size***: body length up to 2 mm (*n* = 4).

***Colour pattern***: ground colour pale. Eyepatch dark blue. Antennae (Ant I–IV) and tibiotarsus with blue pigment (Fig. 3).

***Head***: antenna up to 0.93 mm long, approximately 0.44× body length. Relative lengths of antennal segments I:II:III:IV = 1:1.8:1.2:1.7 (*n* = 4). Distal part of Ant IV with pointed pin chaetae, subapical organite rod-like, and bilobed apical bulb. Eyes 8 + 8, G and H smaller than the others. Labral chaetae formula 4/5, 5, 4, prelabral chaetae apparently smooth, other labral chaetae smooth. Distal margin of labrum with four papillae, each bearing one denticle. Lateral process of labial palp E straight, not reaching the apex of the labial papilla (Fig. 20). Labial triangle chaetae (M_2_RELL) as in Fig. 21, all finely ciliate, with R smaller.

***Scales***: scales of type B (short rib type) *sensu*Zhang et al. (2011), but with longer ridges than previously illustrated forms, intermediate between known variants, with a pointed (slightly rounded) tip not reported in other species (Fig. 22).

***Body and legs***: trochanteral organ with 12–14 smooth spiny chaetae. Claw (Fig. 23) with four inner teeth, one paired at 50% of the inner edge of claw, and two distal unpaired teeth, (similar in length to the paired ones) located at 70% and 80% from the base. Empodium acuminate, with serrated outer edge. Tenent hair thick, clavate, and longer than the inner edge of the claw. Abd IV three times as long as Abd III along the dorsal midline. Mucro with both teeth similar in size (Fig. 24).

**Figures 25–27.**
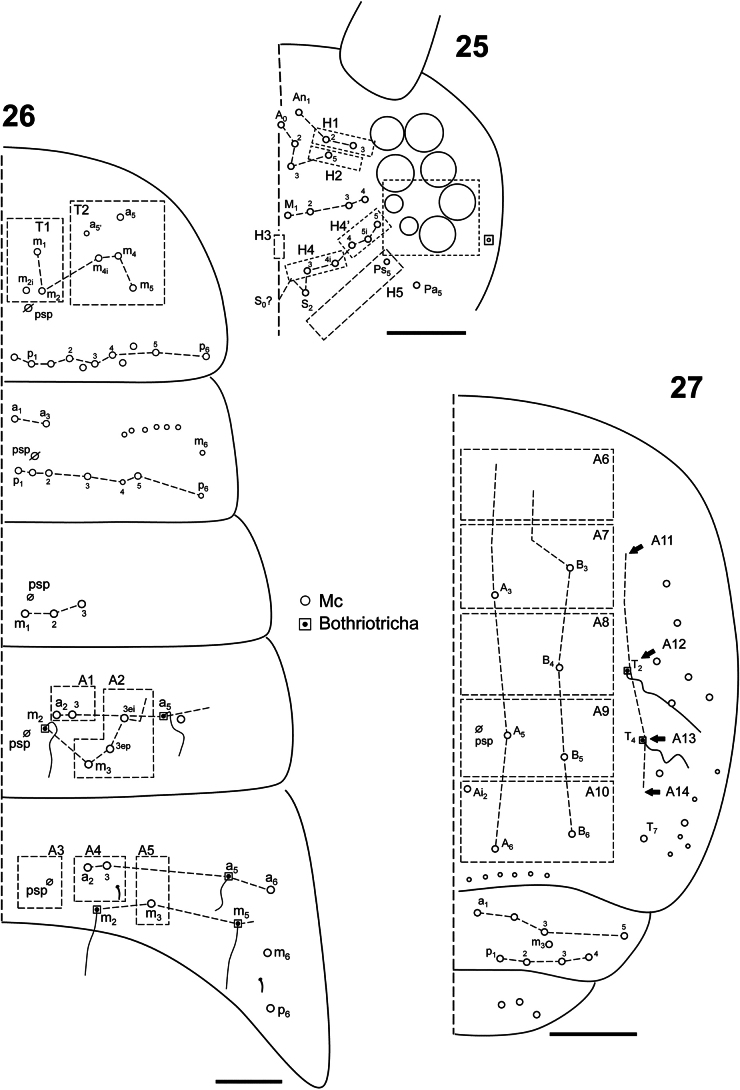
*Willowsia solanella* sp. nov. dorsal macrochaetotaxy. **25**. Head; **26**. Thorax II–III and Abd I–III; **27**. Abdominal IV and partial Abd V. Scale bars: 0.05 mm (**25, 26**); 0.1 mm (**27**).

***Macrochaetotaxy***: dorsal cephalic chaetotaxy (Fig. 25) with An_1–3_, A_0–3_, A_5_, M_1−4_, S_0_?, S_2–3_, S_4i_, S_4_, S_5i_, S_5_, Ps_5_, and Pa_5_ as macrochaetae (Mc.) Th II with dorsal macrochaetae: m_1_, m_2_, and m_2i_; medio-median: Mc a_5_ (with a_5’_ as Mc or big mes), m_4_, m_4i_, and m_5_; and 10 medio-lateral posterior Mc on each side. Specialized chaetae (S-chaetae) on the antero-lateral margin not observed. Th III with two median anterior Mc, six antero-lateral Mc on each side and seven Mc on the posterior margin. Abd I with three Mc (m_1–3_). Abd II with five Mc (a_2–3_, m_3_, m_3e_ and m_3ea_) and one lateral Mc (m_5_) lateral; S-chaetae not observed. Abd III with three dorso-central Mc (a_2–3_, m_3_), and three lateral Mc (a_6_, m_6_ and p_6_); S-chaetae present but microsensillum (ms) not observed (Fig. 26). Abd IV with Ai_2_, A_3_, A_5–6_, B_3–6_ as Mc (Fig. 27). General chaetotaxy formula: 2-1-0-2-1a/3-4/2-3/0-2-1/0-2-1-2-3 (after Jordana and Baquero 2005).

##### Etymology.

The specific epithet *solanella* is a diminutive derived from *Solanum tuberosum* Linnaeus, 1753 (potato), referring to the microhabitat in which the species was discovered.

##### Ecology.

The holotype and paratypes were collected from a cardboard box containing potatoes in a domestic environment. Slight humidity and localized fungal growth created favourable conditions, providing both moisture and nutritional resources (fungal hyphae, bacteria, and decaying plant material).

*Willowsia solanella* sp. nov. appears to be a synanthropic species inhabiting indoor environments where moisture, organic particles, and fungal growth are present. Like other members of the genus *Willowsia*, it is fungivorous–detritivorous, feeding primarily on superficial molds and fine organic debris. Its presence is not associated with damage to stored products and should be considered ecologically harmless, likely contributing to the decomposition of organic matter in human-modified microhabitats.

##### Remarks.

The genus *Willowsia* Shoebotham, 1917 (Collembola, Entomobryidae) is characterized by the presence of scales and resembles *Entomobrya* in most other morphological traits. Species of *Willowsia* are widely distributed and occupy diverse habitats, including forest litter, bark surfaces, and anthropogenic environments. Several species are strongly synanthropic, frequently occurring in buildings, warehouses, and storage facilities where humidity and organic debris are present (Cipola and Katz 2021).

In Europe, including Romania, the genus is represented by relatively few species, with only three currently known: *W. buski* (Lubbock, 1870), *W. nigromaculata* (Lubbock, 1873), and *W. platani* (Nicolet, 1842). Despite their wide distribution, taxonomy within the genus remains challenging due to pigmentation variability, incomplete historical descriptions, and limited modern chaetotaxic studies. Recent revisions have clarified species boundaries within the group, however, several undescribed taxa—particularly those from synanthropic environments—remain to be documented.

Among known species, *W. samarkandica* Martynova, 1972, *W. yiningensis* Zhang, Chen & Deharveng, 2011, and *W. zhaotongensis* Chai & Ma, 2017 share the same chaetotaxy in Abd II–III (2-3/0-2-1). However, these species differ in Th II chaetotaxy: four medial Mc and three lateral Mc in *W. samarkandica*, two medial and three lateral in *W. yiningensis*, and three medial and five lateral in *W. zhaotongensis*. Additionally, the number of central abdominal chaetae between trichobothria T2–T4 on Abd IV differs: 32 in *W. samarkandica*, 22 in *W. zhaotongensis*, 14 in *W. yiningensis*, and 16 in *W. solanella* sp. nov. Differences between these species and the new taxon are summarized in Table 3.

## Supplementary Material

XML Treatment for Entomobrya

XML Treatment for Entomobrya cezarae

XML Treatment for Entomobrya antoniae

XML Treatment for Willowsia

XML Treatment for Willowsia solanella

## Additional information

### Artificial Intelligence (AI) use

The authors accept full responsibility for the content of the manuscript, including the disclosure of any use of AI.

No AI tools were used in the preparation of this manuscript.

### Author ORCIDs

R. Jordana https://orcid.org/0000-0001-9088-787X

C. Fiera https://orcid.org/0000-0002-0125-5879

E. Baquero https://orcid.org/0000-0002-2145-8606

### Data availability

All of the data that support the findings of this study are available in the main text.

## References

[B1] Baquero E, Jordana R (2008) Five new species of *Entomobrya* Rondani, 1861 (Collembola, Entomobryidae) from sacred forests of *Juniperus tibetica* near Lhasa (NW China). Soil Organisms 80(1): 1–18.

[B2] Baquero E, Arbea J, Jordana R (2010) A new species of *Entomobrya* from China (Collembola, Entomobryidae). Soil Organisms 82(3): 277–284.

[B3] Baquero E, Mandal GP, Jordana R (2014) Singular fauna of Entomobryidae (Collembola) from “Land of Passes” in the Himalayas, India. Florida Entomologist 97(4): 1554–1587. 10.1653/024.097.0430

[B4] Baquero E, Mandal GP, Jordana R (2015) Entomobryoidea (Collembola) from Himachal Pradesh (India) in the Himalayas. Zootaxa 4027(1): 1–41. 10.11646/zootaxa.4027.1.1 26624165

[B5] Baquero E, Potapov M, Jordana R (2021) New species and records of Entomobryidae and Orchesellidae (Collembola) from the East Caucasus (Russia). Zootaxa 4991(2): 247–270. 10.11646/zootaxa.4991.2.2 34186848

[B6] Bellinger PF, Christiansen KA, Janssens F (1996–2024) Checklist of the Collembola of the World. 10.1016/b978-0-12-374144-8.00064-3

[B7] Börner C (1913) Zur Collembolenfauna Javas. Das trochanteralorgan der Entomobryiden. Tijdschrift Voor Entomologie 56: 44–61.

[B8] Chai R, Ma Y (2017) Two new species of *Willowsia* (Collembola: Entomobryidae) from Yunnan Province, China. European Journal of Taxonomy 311: 1–12. 10.5852/ejt.2017.311

[B9] Christiansen K, Bellinger P (1980) Family Entomobryidae. In: Christiansen KA, Bellinger PE (Eds) The Collembola of North America north of the Rio Grande. A Taxonomic Analysis. Part 3. Grinnell College, Iowa, December 1980, 785–1042.

[B10] Cipola NG, Katz AD (2021) Morphological and molecular analysis of *Willowsia nigromaculata* (Collembola, Entomobryidae, Entomobryinae) reveals a new cryptic species from the United States. European Journal of Taxonomy 739: 92–116. 10.5852/ejt.2021.739.1269

[B11] Deharveng L (1983) Morphologie évolutive des collemboles Neanurinae en particulier de la lignée neanurienne. Travaux du Laboratoire d’Ecobiologie des Arthropodes Edaphiques, Toulouse 4: 1–63.

[B12] Denis JR (1933) Collemboles récoltés par M. P. Rémy en Yougoslavie et en Macédoine grecque (note préliminaire). Bulletin de la Société Entomologique de France 38(14): 211–213. 10.3406/bsef.1933.14608

[B13] Fjellberg A (1999) The labial palp in Collembola. Zoologischer Anzeiger 237: 309–330.

[B14] Gisin H (1967) Espèses nouvelles et lignées évolutives de *Pseudosinella* endogés. Memórias e Estudos do Museu Zoológico da Universidade de Coimbra 301: 5–25.

[B15] Jantarit S, Surakhamhaeng K, Deharveng L (2020) The multiformity of antennal chaetae in *Troglopedetes* Joseph, 1872 (Collembola, Paronellidae, Troglopedetinae), with descriptions of two new species from Thailand. ZooKeys 987: 1–40. 10.3897/zookeys.987.54234 PMC7666072 33223884

[B16] Jordana R (2012) Synopses on Palaearctic Collembola – Capbryinae & Entomobryini. Soil Organisms 84(1): 1–390.

[B17] Jordana R, Baquero E (1999) Redescription of *Entomobrya schoetti* (Collembola, Entomobryidae, Entomobryinae), third record to the world fauna. Boletín de Sanidad Vegetal Plagas 25(1): 99–105.

[B18] Jordana R, Baquero E (2005) A proposal of characters for taxonomic identification of *Entomobrya* species (Collembola, Entomobryomorpha), with description of a new species. Abhandlungen und Berichte des Naturkundemuseum Gorlitz 76(2): 117–134.

[B19] Jordana R, Greenslade P (2020) Biogeographical and ecological insights from Australasian faunas: the megadiverse collembolan genus, *Entomobrya* (Entomobryidae). Zootaxa 4770(1): 001–104. 10.11646/zootaxa.4770.1.1 33055632

[B20] Jordana R, Potapov M, Baquero E (2011) New species of Entomobryini from Russia and Armenia (Collembola, Entomobryomorpha). Soil Organisms 83(2): 221–248.

[B21] Katz AD, Giordano R, Soto-Adames FN (2015) Taxonomic review and phylogenetic analysis of fifteen North American *Entomobrya* (Collembola, Entomobryidae), including four new species. ZooKeys 525: 1–75. 10.3897/zookeys.525.6020 PMC4607850 26487816

[B22] Linnaeus C (1753) Species plantarum, exhibentes plantas rite cognitas, ad genera relatas, cum differentiis specificis, nominibus trivialibus, synonymis selectis, locis natalibus, secundum systema sexuale digestas. Holmiae, Stockholm, Vol. 1, 560 pp.

[B23] Lubbock J (1870) Notes on the Thysanura. – Part IV. The transactions of the linnean society of London 27(2): 277–297. 10.1111/j.1096-3642.1870.tb00214.x

[B24] Lubbock J (1873) Monograph of the Collembola and Thysanura. Royal Society, London, 276 pp. 10.5962/bhl.title.11583

[B25] Martynova EF (1972) Springtails (Collembola) inhabiting the outlets of subterranean water in the Kirghiz and Uzbek SSR. Proceedings of the Zoological Institute of the USSR Academy of Sciences 51: 147−150.

[B26] Nicolet H (1842) Recherches pour Servir á l’histoire des podurelles. Nouveaux Mémoires de la Société Helvétique des Sciences Naturelles 6: 1–88.

[B27] Rondani C (1861) *Entomobrya* pro *Degeeria* Nic. In: Stocche A (Ed.) Dipterologiae Italicae Prodromus. Vol. IV. Tipographia A. Stocchii, Paris, 40. 10.5962/bhl.title.8160

[B28] Santos NMDC, Dos Santos-Costa RC, Siqueira OJR, Godeiro NN, Bellini BC (2020) Two new species of *Entomobrya* Rondani (Collembola, Entomobryidae) from northeastern Brazil and comments on the genus. Zootaxa 4731(1): 043–062. 10.11646/zootaxa.4731.1.3 32229827

[B29] Shoebotham J (1917) Notes on the Collembola.—Part 4. The classiﬁcation of the Collembola; with a list of genera known to occur in the British Isles. Annals and Magazine of Natural History 19(114): 425–436. 10.1080/00222931709486959

[B30] Soto-Adames FN (2008) Postembryonic development of the dorsal chaetotaxy in *Seira dowlingi* (Collembola, Entomobryidae); with an analysis of the diagnostic and phylogenetic significance of primary chaetotaxy in *Seira*. Zootaxa 1683: 1–31. 10.11646/zootaxa.1683.1.1

[B31] Stach J (1922) Apterygoten aus dem nordwestlichen Ungarn. Annales Musei Nationalis Hungarici 19: 1–75.

[B32] Stach J (1963) The Apterygotan fauna of Poland in relation to the world-fauna of this group of insects. Tribe: Entomobryini. Polska Akademia Nauk, Kraków: 1–126.

[B33] Szeptycki A (1979) Chaetotaxy of the Entomobryidae and its phylogenetical significance. Morpho-systematic studies on Collembola. IV. Polska Akademia Nauk, Zakład Zoologii Systematycznej i Doświadczalnej, Państwowe Wydawnictwo Naukowe, Warszawa–Kraków, 219 pp.

[B34] Tömösvary O (1882) Adatok Hazánk Thysanure-Faunájához. A Mathematikai és Természettudományi Osztályok Közlönye 18: 119–130.

[B35] Viana SDS, de Morais JW, Cipola NG (2024) Taxonomic revision of *Entomobrya* Rondani, 1861 (Collembola: Entomobryidae: Entomobryinae) from the Brazilian Amazon. Zootaxa 5452(1): 001–110. 10.11646/zootaxa.5452.1.1 39646231

[B36] Zhang F, Chen JX, Deharveng L (2011) New insight into the systematics of the *Willowsia* complex (Collembola: Entomobryidae). Annales de la Société Entomologique de France 47(1−2): 1−20. 10.1080/00379271.2011.10697692

